# Scaffolds Formed via the Non-Equilibrium Supramolecular Assembly of the Synergistic ECM Peptides RGD and PHSRN Demonstrate Improved Cell Attachment in 3D

**DOI:** 10.3390/polym10070690

**Published:** 2018-06-21

**Authors:** San-Seint S. Aye, Rui Li, Mitchell Boyd-Moss, Benjamin Long, Sivapriya Pavuluri, Kiara Bruggeman, Yi Wang, Colin R. Barrow, David R. Nisbet, Richard J. Williams

**Affiliations:** 1 Center for Chemistry and Biotechnology, Deakin University, Waurn Ponds, VIC 3217, Australia; aye.sanseint@gmail.com (S.-S.S.A.); liruihn@163.com (R.L.); bm.long@federation.edu.au (B.L.); Colin.barrow@deakin.edu.au (C.R.B.); 2 School of Engineering, RMIT University, Bundoora, VIC 3083, Australia; s3383061@student.rmit.edu.au; 3 Biofab3D, St. Vincents’ Hospital, Fitzroy, VIC 3000, Australia; 4 Faculty of Science and Technology, Federation University, Mt. Helen, VIC 3350, Australia; 5 School of Medicine, Deakin University, Waurn Ponds, VIC 3217, Australia; pspriyamurthy@gmail.com; 6 Research School of Engineering, Australian National University, Canberra, ACT 0200, Australia; Kiara.Bruggeman@anu.edu.au (K.B.); Yi.Wang@anu.edu.au (Y.W.)

**Keywords:** self-assembly, hydrogel, peptides, cell adhesion

## Abstract

Self-assembling peptides (SAPs) are a relatively new class of low molecular weight gelators which immobilize their solvent through the spontaneous formation of (fibrillar) nanoarchitectures. As peptides are derived from proteins, these hydrogels are ideal for use as biocompatible scaffolds for regenerative medicine. Importantly, due to the propensity of peptide sequences to act as signals in nature, they are easily functionalized to be cell instructive via the inclusion of bioactive epitopes. In nature, the fibronectin peptide sequence, arginine-glycine-aspartic acid (RGD) synergistically promotes the integrin α_5_β_1_ mediated cell adhesion with another epitope, proline-histidine-serine-arginine-asparagine (PHSRN); however most functionalization strategies focus on RGD alone. Here, for the first time, we discuss the biomimetic inclusion of both these sequences within a self-assembled minimalistic peptide hydrogel. Here, based on our work with Fmoc-FRGDF (*N*-flourenylmethyloxycarbonyl phenylalanine-arginine-glycine-aspartic acid-phenylalanine), we show it is possible to present two epitopes simultaneously via the assembly of the epitopes by the coassembly of two SAPs, and compare this to the effectiveness of the signals in a single peptide; Fmoc-FRGDF: Fmoc-PHSRN (*N*-flourenylmethyloxycarbonyl-proline-histidine-serine-arginine-asparagine) and Fmoc-FRGDFPHSRN (*N*-flourenylmethyloxycarbonyl-phenylalanine-arginine-glycine-asparticacid-phenylalanine-proline-histidine-serine-arginine-asparagine). We show both produced self-supporting hydrogel underpinned by entangled nanofibrils, however, the stiffness of coassembled hydrogel was over two orders of magnitude higher than either Fmoc-FRGDF or Fmoc-FRGDFPHSRN alone. In-vitro three-dimensional cell culture of human mammary fibroblasts on the hydrogel mixed peptide showed dramatically improved adhesion, spreading and proliferation over Fmoc-FRGDF. However, the long peptide did not provide effective cell attachment. The results demonstrated the selective synergy effect of PHSRN with RGD is an effective way to augment the robustness and functionality of self-assembled bioscaffolds.

## 1. Introduction

The self-assembly of biomolecules have received significant attention as a facile route to fabricate bioinspired scaffolds with porous, three-dimensional (3D) architectures, that have increasingly well-understood mechanisms to control their formation [1,2,3]. Nevertheless, for effective cell culture and tissue growth, significant functionalization is required to truly exploit their promise as biomaterials; the engineering of biomaterial scaffolds should to not only mechanically support cells, but also actively modulate cellular activities [4,5,6]. Recently, our research has focused on developing synthetic low molecular weight peptides which contain biologically relevant functional groups [7]. The assembly of these molecules yields hydrogels by immobilizing the solvent via the formation of ordered nanostructured architectures with a high density of bioactive signals, thereby allowing the potential for control over cell behaviors [8]. Self-assembled peptide scaffolds are useful synthetic scaffolds for biomedical and tissue engineering applications, as they have the potential to mimic the morphological and chemical nature of the extracellular matrix. These scaffolds have generally been functionalized by including a specific amino acid sequence inserted synthetically into the peptide backbone. However, due to length constraints, these scaffolds have been typically limited to a single sequence, and further functionalized with macromolecules [9].

In nature, the scaffold responsible for this level of control over cell fate is the tissue-specific extracellular matrix (ECM); a multifunctional apparatus that provides structural support and dynamic cellular signaling to control a range of cellular functions, such as cell adhesion, differentiation, migration and proliferation [10]. Generally, cell adhesion is mediated by the specific interactions between cell surface adhesion receptors and specific amino-acid sequences, termed epitopes, presented in specific densities and conformations by ECM proteins. In particular, cell adhesive interactions play a major role during multiple normal physiological process such as embryonic development and wound repair, and also during the progression of diseases such as cancer [11]. Fibronectin (FN) is well characterized ECM protein; chiefly due to its ability to mediate the adhesion and spreading of various cell types through the organization of homologous repeating modules into functional domains [12]. The most widely studied epitope derived from fibronectin (FN) is the tripeptide sequence arginine-glycine-aspartic acid (RGD). Significant research efforts have employed the RGD epitope as a biofunctional adhesive site to functionalize materials [13,14]. It has been suggested however, that RGD alone cannot accurately mimic the affinity of FN for integrins [15,16]. This limitation arises because FN presents a second epitope, proline-histidine-serine-arginine-asparagine (PHSRN), that operates synergistically with RGD to ensure effective cellular response via activation of the α_5_β_1_ receptor [17,18]. Recently, the interest in presenting both the RGD and PHSRN epitope has grown due to a demonstrated ability to enhance cell adhesion, migration, and spreading [19,20], as well as osteoblast differentiation [14,21,22] and angiogenesis [23]. These studies have demonstrated that effective functionalization of hydrogels is achieved with the two epitopes separated by a spacer—such as polyglycine (G_n_); RGDG_13_PHSRN within a polyethylene glycol hydrogel [24] or a serine-glycine spacer (GGGSSPHSRN(SG)_5_RGDSP) in a self-assembling peptide-amphiphile (PA) [25]. These molecules however are large and synthetically challenging. Advancement would be to achieve the same improvement in cell response in minimally designed systems. Recently efforts have been made to form hydrogels by mixing two or more self-assembling peptide derivatives together to control their mechanical and morphological properties, introduce functional signals, and yield two-component hydrogels from sequences that do not assemble independently. Therefore, we hypothesized that by distributing RGD and PHSRN in a two-component system, we could create a minimalistic cell culture scaffold using very short peptide sequences. The combination of multiple signals in a single, homogenous scaffold offers the potential for a synergistic effect on cellular response through the improved spatial and chemical arrangement of multiple bioactive signals [26]. Hydrogels from multiple components have been reported using coassembly, mixing, and self-sorting [28]. The mechanism by which the SAPs molecularly pack into the fibres important to the function/design parameters remains a challenge [27]. Systems have been reported where multiple bioactive epitopes have been investigated in PA systems, with limited success [28], and with doping a Fmoc-RGD molecule to functionalize fibrils of Fmoc-FF [13]. We have recently published on the assembly of functional Fmoc-SAPs from separate ECM proteins [26]. We report here on the coassembly of two synergistic epitopes required to mimic the function of a single ECM protein.

In this work, we designed three peptide sequences containing one or both of the synergistic bioactive epitpopes: Fmoc-FRGDF, Fmoc-PHSRN and Fmoc-FRGDFPHSRN. The self-assembly mechanism, mechanical properties, micro- and nano-structures of the peptide scaffolds were evaluated and compared, and the synergistic effect of peptides within the scaffolds was assessed by monitoring the adhesion, spreading and proliferation of human mammary fibroblast cells (HMFCs).

## 2. Experimental Section

### 2.1. Peptide Synthesis

The synthesis of all Fmoc-peptides was performed as previously reported [8]. Purity of each Fmoc-Peptide was >95% as determined by reverse phase high performance liquid chromatography.

### 2.2. Hydrogel Formation

The required amount of Fmoc-peptides was suspended into 400 µL Mili-Q water (purified by Mili-Q Advantage A10 System, Merck Milipore, Melbourne, Australia). 0.5 M NaOH (Sigma-Aldrich Pty. Ltd., Sydney, Australia) was added dropwise to the aqueous suspension of the peptides until fully dissolved. During this time, the peptide solutions were placed in an ultrasonicator (Soniclean Pty. Ltd., Thebardson, Australia) and vortexed interchangeably. A required volume of 0.1 M HCL was then added drop wise until physiological pH (7.4) was reached with vortexing. Finally, 0.1 M phosphate buffer saline (PBS) (pH 7.4) was added to the peptide solution to bring total volume up to 1 mL and maintain pH, then kept at room temperature for gelation (total peptide concentration 1 wt %).

### 2.3. Circular Dichroism (CD)

Information on secondary structure of the Fmoc-peptides was obtained by CD spectra using a Jasco J-815 spectropolarimeter using a 1-mm path length quartz cell (Starna Pty. Ltd., Castle Hill, Australia). Measurements were carried out in continuous scanning mode, at a scan rate of 50 nm/min with a bandwidth of 1 nm and 2 s integration time. Reported spectra were averages of 10 scans with data-smoothing, collected over a range of wavelengths from 190 to 300 nm. Sample was diluted 20 times using Mili-Q water to give a peptide concentration of 0.05 mg/mL. Mili-Q water was used as a background and subtracted from all spectra.

### 2.4. Fourier Transform Infrared Spectroscopy (FT-IR)

FT-IR spectra were collected on a Nicolet 6700 FTIR spectrophotometer (Watham, MA, USA) in attenuated total reflection (ATR) mode. 0.1 M PBS was used as a background before samples measurement. 12 µL of peptide hydrogel was spread directly on the ATR crystal and allowed to dry for 10 min evaporating the solvent which enables minimal contribution form the solvent to the spectra. The samples were scanned between the wavenumbers of 4000 and 400 cm −1 over 64 scans. Data analysis was carried out using OPUS software (Preston, Victoria, Australia).

### 2.5. Fluorescence Spectroscopy

Fluorescence emission spectra were analysed on a Cary Eclipse fluorescence spectrophotometer (Agilent Technologies, Santa Clara, CA, USA). Samples were diluted using Mili-Q water to give a final peptide concentration of 0.5 mg/mL. A quartz cuvette of 1 mm path length (Starna Pty. Ltd., Castle Hill, Australia) was used. Excitation wavelength were at 250 nm and emission data were collected between 300 and 600 nm with a data pitch of 1.0 nm and a scanning speed of 600 nm/min.

### 2.6. Atomic Force Microscopy (AFM)

AFM images were obtained using a Multimode 8 AFM (Bruker BioSciences Corporation, Billerica, MA, USA) in peak force QNM (quantitative nanomechanical mapping) mode. Samples were diluted 20 times using Mili-Q water to make a final concentration of 0.05 mg/mL. A 15 µL aliquot of the diluted solution was deposited onto a highly ordered pyrolytic graphite (HOPG) substrate (SPI), the excess sample solution was absorbed by pipettes and left to dry overnight. Scanasyst-air silicon-nitride tip (Bruker) with spring constant of 0.4 N/m was used. AFM scans were taken at 512 × 512 pixels resolution and the topographic images of the samples were captured at a scan rate of 0.93 Hz with scan size of 10 µm.

### 2.7. Transmission Electron Microscopy (TEM)

TEM visualization was operated on a JEOL-2100 LaB6 transmission electron microscopy (JEOL Ltd., Tokyo, Japan) connected to a Gatan Orius CCD camera (Cuddy, PA, USA) at an operation voltage of 100 kV. For effective penetration of electron beam, the hydrogel sample was diluted 5 times (peptide concentration 0.2 mg/mL) and 12 µL droplet of diluted solution was placed on an agar lacey carbon coated film on 300 mesh copper grids (Emgrid Pty. Ltd., Gulf View Hieghts, Australia) and allowed to adsorb for 30 s, the excess fluid was blotted down using Whatman filter paper (No. 1). In negative staining, the carbon side of the grid was stained with one drop of NanoVan (Bio-Scientific Pty. Ltd., Yaphank, NY, USA). After 5 min, the excess fluid was removed from the grid and dried in air for 2 min with the carbon side up; lastly the grid was put into grid box to leave it dry overnight.

### 2.8. Small-Angle X-ray Scattering

SAXS was performed using the SAXS/WAXS beamline at the Australian Synchrotron (Melbourne, Australia). Measurements were taken at a calibrated camera length of 1598 mm with an X-ray energy of 11 KeV (1.12713 Å). This camera length allowed for the scattering vector (*q*) to be measured across the range of 0.01 to 0.6 Å^−1^. The diffraction pattern was recorded on a Pilatus 1M detector (170 mm × 170 mm, effective pixel size of 172 μm × 172 μm), and processed using the Australian Synchotron ScatterBrain Software (Melbourne, Victoria, Australia). Hydrogels were prepared as detailed above two days prior and loaded into 1.5 mm sealed glass capillaries. PBS backgrounds were collected before samples were loaded. Each triplicate sample (and background) was subjected to three 5-s exposure times at multiple positions along the capillary. Repeat measurements were summed using Scatterbrain and *q* calibrated using an AgBeh sample. The intensity was normalized and set on an absolute scale using water and air shots. Due to poor scattering, backgrounds were scaled by 0.9 prior to subtraction from the sample scattering data. The data was then subjected to indirect Fourier transform (IFT) analysis and P(r) inversion using SASView (SASView, Victoria, Australia) to calculate the average diameter of the fibrils in the sample.

### 2.9. Oscillatory Rheometry

To investigate the mechanical properties of the hydrogels, rheological measurements were taken on a Discovery Hybrid Rheometers (TA Instruments, New Castle, DE, USA) using a cone-plate geometry (40 mm, 2°1′37′′) with a 51 µm truncation gap. About 1 mL of Fmoc-peptide hydrogels were placed onto the lower plate to completely cover the measured area. To ensure the measurements were made in the linear viscoelastic regime, amplitude sweeps were performed at a constant frequency of 10 rad/s with shear strain 0.01–100%, where no variation in elastic modulus *G*’ and viscous modulus *G*” was observed up to a strain of 1% for all peptide samples. Data on dynamic frequency sweeps were collected over a range between 0.1 and 100 rad/s at a constant strain of 0.4%. Temperature was maintained at 25 °C via the use of Peltier plate control. A water trap was used to minimize evaporation from the hydrogel. All measurements were carried out in triplicate to ensure reproducibility with the show of the average data.

### 2.10. Cell Culture

Primary HMFCs were obtained from ScienCell Research Laboratories. Cells were cultured as described previously [8]. Briefly, cells were maintained and cultured in the fibroblast media containing 10% fetal bovine serum, fibroblast growth supplement, 2% penicillin/streptomycin (ScienCell Research Laboratories, Carlsbad, CA, USA) at 37 °C. Prepared peptide hydrogels (40 µL) were added to each well of 96-well plate (Corning Inc., Corning, NY, USA). Hydrogels were allowed to solidify at 37 °C for 10 days.

### 2.11. Cell Proliferation Assay (MTS Assay)

HMFC cells (4000 per well in 100 µL of media) were cultured on the hydrogels constitutively for 24, 48 and 72 h on the hydrogels containing peptides, Fmoc-FRGDF, Fmoc-FRGDFPHSRN, and peptide mixture of Fmoc-FRGDF/Fmoc-PHSRN (1:1, *w*/*w*). Quantification of live cells was performed based on colorimetric method by adding MTS reagent (Promega, Madison, WI, USA) to the cells growing on hydrogels every 24 h. MTS (20 µL) was added to the cells and mixed gently. Further the cells were incubated for 4 h at 37 °C before reading of absorbance values at 490 nm using spectrophotometer (Bio-Rad, Hercules, CA, USA).

### 2.12. Actin Staining

After 48 h of culture, cells growing on the hydrogels were fixed using 4% paraformaldehyde for 10 min at room temperature. Cells were further washed gently with 1X PBS twice followed by permeabilization treatment with 0.1% triton X-100 for 15 min at room temperature. Cells were carefully washed twice with 1X PBS without disturbing the gels. Rhodamine phalloidin (Life Technologies, Mulgrave, Victoria, Australia) was used to stain the actin according to manufacturer’s instructions. Stained cells were observed under the fluorescent microscope (Nikon, Tokyo, Japan). ImageJ analysis (Bethedsa, MD, USA) was used to analyse the area and intensity of the staining as per the software instructions. Graph pad prism (version 3.03, San Diego, CA, USA) was used to calculate the significant student t-test for the values obtained using ImageJ.

## 3. Results and Discussion

**Figure 1:**
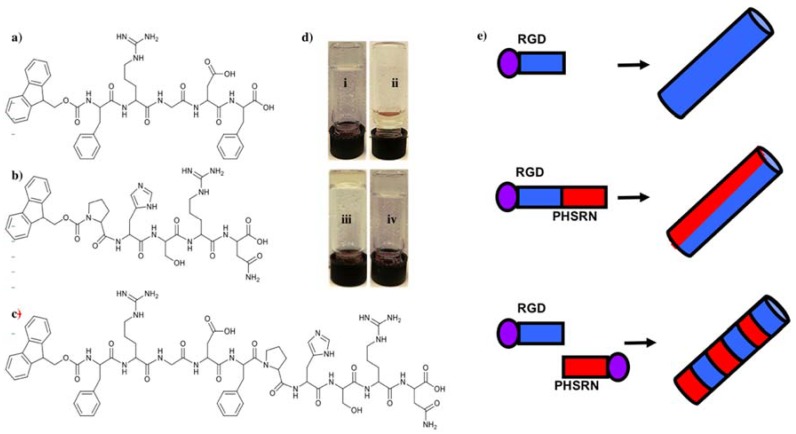
Molecular structure of three Fmoc-derived peptides. (**a**) Fmoc-FRGDF; (**b**) Fmoc-PHSRN; (**c**) Fmoc-FRGDFPHSRN and (**d**) hydrogels formed of (i) Fmoc-FRGDF; (ii) Fmoc-PHSRN; (iii) Fmoc-FRGDFPHSRN and (iv) peptide mixture of Fmoc-FRGDF/Fmoc-PHSRN (1:1, *w*/*w*). (**e**) Cartoon schematic of the assembly process.

Three minimalist Fmoc-SAPs, Fmoc-FRGDF, Fmoc-PHSRN and Fmoc-FRGDFPHSRN (Figure 1a–c) were synthesized using a traditional solid phase peptide synthesis (SPPS) method. All of the three peptides formed crystal powders. Next, a peptide mixture of Fmoc-FRGDF/Fmoc-PHSRN (1:1, *w*/*w*) was prepared. These four groups of peptides were then used to form hydrogels using a well-established pH switch method [29]. Our previous research has shown that the pentapeptide Fmoc-FRGDF has the ability to form a clear hydrogel [30,31]. Our experiments confirm that Fmoc-PHSRN alone could not form a hydrogel in all tested conditions (Figure 1d). This inability to form a hydrogel thereby rendered it unavailable as a 3D scaffold, and as such, was discounted for cell studies. This phenomenon may be due to the presence of proline, which previous research has shown cannot form the required hydrogen bonds needed to form stable α-helixes or β-sheets as a result of its cyclic structure, thus lacking the normal NH_2_ backbone [32,33]. It took 10 days to form a stable hydrogel for Fmoc-FRGDFPHSRN, as opposed to 3 days for the Fmoc-FRGDF/Fmoc-PHSRN coassembly. The delay of hydrogel formation could be due to the disruptive influence of the rigid proline within the SAP backbone, and may explain the limited research to date using this sequence as an assembly motif.

However, when the materials were allowed to form a self-supporting hydrogel for 10 days, spectroscopic analysis of the Fmoc-SAP systems confirmed that the assembly is consistently driven by a similar mechanism, where Fmoc- interactions, stabilized by a range of hydrogen bonds in a hierarchical assembly [34], supported by the structural similarities observed between the nanofibrous networks [35].

**Figure 2:**
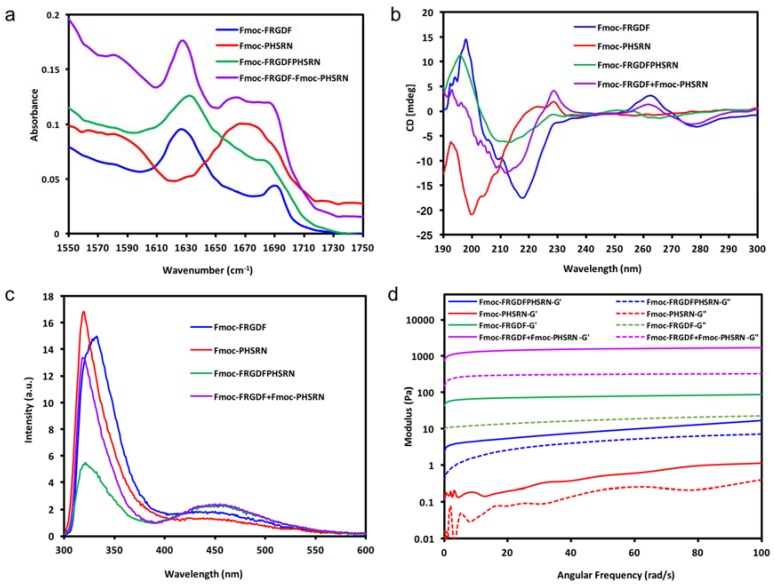
Spectroscopic and rheometer anaylsis for the four peptide hydrogel groups. (**a**) Truncated FT-IR absorption spectra in amide I region; (**b**) CD spectra; (**c**) fluorescence spectra; (**d**) rheometry curves.

In each case, the FTIR and CD spectra indicate the organization of the molecules into a dominant structural ordering. Fourier transform infrared spectroscopy (FT-IR) was used to determine the dominant organization of the peptide backbone. As shown in Figure 2a, Fmoc-FRGDF, Fmoc-FRGDFPHSRN and Fmoc-FRGDF/Fmoc-PHSRN showed two distinct absorption peaks in amide I region at 1627 cm^−1^ However, in Fmoc-PHSRN alone, there was no evidence of a β-sheet structure around 1630 cm^−1^. Instead, an absorption peak with a maximum at 1667 cm^−1^ was detected, which represents random coil structure [36] For the Fmoc-FRGDF/Fmoc-PHSRN peptide mixture, another peak centered at 1664 cm^−1^ was observed, indicating that in addition to the core of the β-sheet structure, other secondary structures predominantly composed of an element of random coil suggesting that the spatial requirements for the packing of the two peptides, particular those including proline, is not ideal. The peak at 1664 cm^−1^ is absent in the spectra of Fmoc-FRGDF peptide; it is suggested therefore, that conformational constraints introduced by the Fmoc-PHSRN peptide have an impact on the β-sheet based supramolecular structure formation in Fmoc-FRGDF.

Circular dichroism (CD) was used for the analysis of secondary structures of amino acids in conjunction with FT-IR. The CD spectrum (Figure 2b) of Fmoc-FRGDF displayed a negative peak at 218 nm, which shows the Cotton effect induced by n–π* transition, and a positive peak at 198 nm indicates π–π* transition [37]. The two distinct peaks demonstrate the β sheet formation pattern [37], reinforcing the results noted in the FT-IR. Another positive peak appearing at 263 nm is likely from the induced chirality of aromatic stacking moieties, particularly the Fmoc-group placed in the environment of a supramolecular assembly [38]. CD spectrum of the non-gelator Fmoc-PHSRN has a strong negative band at 200 nm and a weaker positive band at 223 nm, this was likely to adopt a non-hydrogen bonded polyproline type II (PPII) helical structure and present as random single units in dilute condition (10 mg/mL) [39,40], which is concurrent with FT-IR data. With PPII confirmation and random coils, the peptide remains very flexible, as can be seen through its fluidic state. The CD spectra of Fmoc-FRGDFPHSRN followed the characteristic of a β-sheet structure with a positive peak centered at 196 nm and a negative peak at 213 nm. For the peptide mixture, the positive peak at 218 nm was not evident while a negative peak centered at 212 nm was observed, which shows an indistinct β-sheet structure, this may be due to the presence of random coil structure as indicated in FT-IR spectrum. The peak observed at ~260 nm in samples of the peptide mixture also shows stacking relating to the Fmoc-group [38]. The presence of a β-sheet structure shows that despite the inability of the proline containing sequence to form β-sheets [33], the rest of the peptide moieties still have the tendency to maintain the β-sheet structure.

Fluorescence spectroscopy has been used by many researchers to monitor the environment of the fluorenyl group [29,41] Excitation peaks at ~330 nm are attributed to Fmoc-peptide monomers [41]. Figure 2c shows that the peak (at ~330 nm) observed for Fmoc-PHSRN was the most intense, indicating that there are more monomers existent in its fluid solution. However, in the gel state Fmoc-FRGDF and Fmoc-FRGDF/Fmoc-PHSRN mixture hydrogel, the intensity of the peak decreased, likely due to hydrogel formation. Fmoc-FRGDFPHSRN showed a much weaker peak at ~330 nm in comparison to the other three peptide solutions, showing that the hydrogel has the least peptide monomers. All peptide groups had broad peaks centered at around 450 nm, indicating excimer formation owing to extensive aromatic stacking interactions (J-aggregates), contributed by phenyl rings of phenylalanine as well as fluorenyl rings [42]. Specifically, multiple fluorenyl rings stacked efficiently in the hydrogel via π–π interactions upon fibrillization. The excimer peak was more pronounced in Fmoc-FRGDF/Fmoc-PHSRN and Fmoc-FRGDFPHSRN hydrogels compared to that of Fmoc-FRGDF, suggesting that the Fmoc-stacking is greatly extended in these peptides. Despite the presence of some random coils in the CD and FT-IR spectra of Fmoc-FRGDF/Fmoc-PHSRN and Fmoc-FRGDFPHSRN peptide hydrogels, the π-stacking interactions between Fmoc-units are still maintained. However, the peak at this wavelength of Fmoc-PHSRN is barely evident, which shows that there is no apparent J-aggregate formation in Fmoc-PHSRN due to the steric effect of proline residue [33] and their orientation within the self-assembled structure, which might not allow proper arrangement of fluorenyl groups for effective π–π stacking interactions. It is likely that the aromatic Fmoc-groups were not aligned in close proximity for these interactions to occur in the nongelator peptide Fmoc-PHSRN. The intensity of the peak at 450 nm in the Fmoc-FRGDF/Fmoc-PHSRNmixture is of greater intensity than that of Fmoc-PHSRN alone, and therefore it can be assumed that weak hydrogen bonding is compensated by stabilization of the Fmoc-environment in the process of gelation.

**Figure 3:**
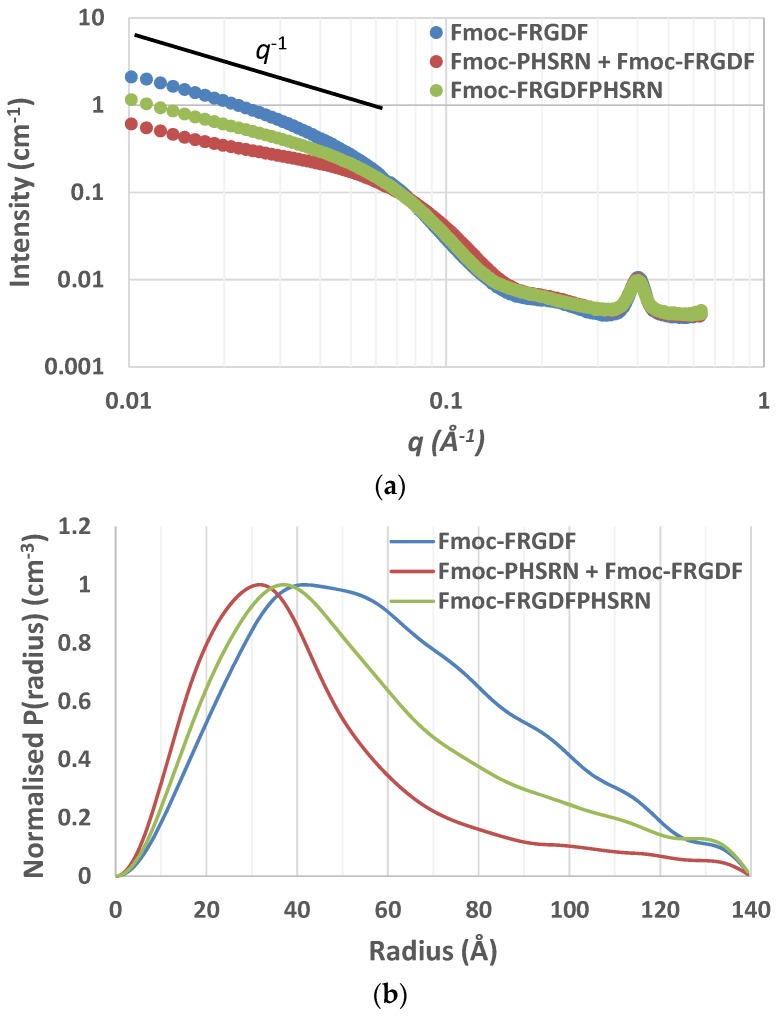
(**a**) SAXS scattering curve of SAPs Fmoc-FRGDF and Fmoc-FRGDF/Fmoc-PHSRN displaying *q*^−1^ relationship at low *q* angles. (**b**) P(r) inversion plot of SAPs Fmoc-FRGDF and Fmoc-FRGDF/Fmoc-PHSRN.

Small-angle X-ray scattering (SAXS) curves supported the cylindrical nature of the nanofibrils as scattering curves for Fmoc-FRGDF, Fmoc-FRGDF/Fmoc-PHSRN, and Fmoc-FRGDFPHSRN provided a *q*^−1^ dependence at low *q* indicating elongated cylindrical structures (Figure 3a). As determining fibril length was outside the *q*-range of SAXS analysis our focus was attuned to determining the average radius. To achieve this measurement the indirect Fourier transform (IFT) method to was used. As the nanostructure was determined to be fibrillar in nature from microscopy the maximum P(r) value given from the IFT calculations was attributed to the average radius of the fibrils. These calculations indicated average fibril diameters of 8.4 nm (*r* = 41.8 Å, σ = 0.7 Å) for Fmoc-FRGDF and 6.3 nm (*r* = 31.3 Å, σ = 1.2 Å) for Fmoc-FRGDF/Fmoc-PHSRN (Figure 3b). The diameters obtained by IFT correlate well to the measurements taken by TEM and correlate well to literature values for fmoc-FRGDF fibril diameter. As spherical precipitates were visualized by TEM in the Fmoc-FRGDFPHSRN sample, the true fibril diameter may be convoluted in IFT analysis. However, IFT provided a maximum P(r) at *r* = 35.3 Å (σ = 1.2 Å) indicating a diameter of 7.1 nm, which is consistent with TEM analysis of the fibrils.

Once the broad self-assembly mechanism was understood, the mechanical properties of the samples was determined by parallel-plate rheometry, as shown in Figure 2d. It can be seen that there is a clear dominance of elastic moduli (*G*’) over corresponding viscous moduli’ (*G*”) of Fmoc-FRGDF, Fmoc-FRGDFPHSRN and the Fmoc-FRGDF/Fmoc-PHSRN mixture, suggesting that all three samples are viscoelastic in nature and behave as typical hydrogels. Alternatively, Fmoc-PHSRN (which can be seen as a viscous liquid in Figure 1) exhibits an extremely low value of *G*’, (~0.5 Pa at 50 rad/s). The structural contribution seen via spectroscopic analysis leads to a value of *G*’ greater than *G*”, indicating the structural contribution of the micelle forming properties of Fmoc-PHSRN. In order to stabilse PHSRN within the network, we utilized two strategies; combining the sequence PHSRN with Fmoc-FRGDF in a single compound to give Fmoc-FRGDFPHSRN, and the coassembly of Fmoc-FRGDF and Fmoc-PHSRN into a single assembly. Fmoc-FRGDF forms a robust, soft hydrogel with a *G*’ value (~50 Pa, 50 rad/s) compared with Fmoc-FRGDFPHSRN (*G*’ ~8.5 Pa, 50 rad/s). Surprisingly, with the addition of the nongelator peptide (Fmoc-PHSRN) to Fmoc-FRGDF in the peptide mixture, the hydrogel is quite robust with a *G*’ value of ~1600 Pa (50 rad/s), exceeding that of Fmoc-FRGDF alone by over two orders of magnitude, clearly demonstrating a much stronger gel than that of Fmoc-FRGDF alone.

**Figure 4:**
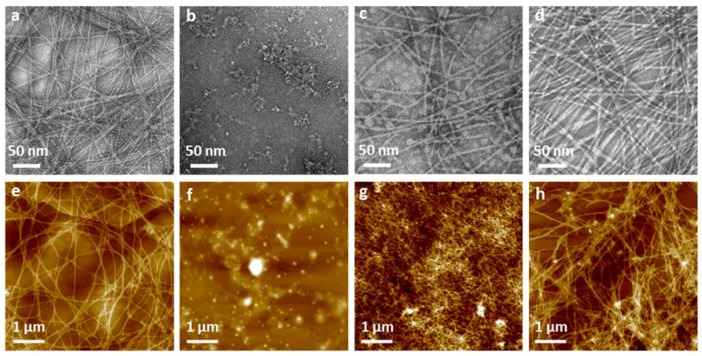
Nano- and microstructure of hydrogels (top panel TEM images and bottom panel AFM images). (**a**,**e**) Fmoc-FRGDF; (**b**,**f**) Fmoc-PHSRN; (**c**,**g**) Fmoc-FRGDFPHSRN and (**d**,**h**) peptide mixture of Fmoc-FRGDF/Fmoc-PHSRN (1:1, *w*/*w*).

Both TEM and AFM were used to assess the nano- and microtopography of the fibers. Apart from nongelator peptide Fmoc-PHSRN, all gel-forming peptides were observed to present 5–10 nm nanofibils in diameter calculated by ImageJ, and further formed fibrillar networks (Figure 4a–d). In TEM images, the Fmoc-FRGDF hydrogel shows a highly ordered nanofibrous network through lateral association of the several fibers and form bundles as seen in Figure 4a. However, no fibrillar structures were seen in the nongelator peptide Fmoc-PHSRN, instead granular particles and clusters were observed (Figure 4b). Since there is no 3D fibrous network to trap water, it is unsurprising that hydrogels were not formed by this peptide at any pH conditions. Long peptide Fmoc-FRGDFPHSRN formed short fibrils and of which no bundled organization of ribbons is seen, unlike that of the other hydrogel samples (Fmoc-FRGDF and Fmoc-FRGDF/Fmoc-PHSRN) (Figure 4c). These truncated nanofibers can explain why it took a considerable amount of time to form gel, as such fibril structures were not efficient enough to immobilize water to form a gel. Some globular structures were also found to coexist with the dispersed fibers (Figure 4c), these structures are probably the unassembled peptide particles. The mixed Fmoc-FRGDF/Fmoc-PHSRN hydrogel showed a well-ordered fibrous network, furthermore, ribbons were observed formed by single fibrils (Figure 4d).

The AFM images revealed well-ordered networks formed in Fmoc-FRGDF (Figure 4e) as was already established in TEM images, this further demonstrates the highly structured nature of the Fmoc-FRGDF hydrogel. Similar to TEM images, AFM images of Fmoc-PHSRN show no fibril formation, rather peptide aggregates are visible (Figure 4f), these are likely due to PPII structures and random coils. In AFM images of Fmoc-FRGDFPHSRN, a dense network existing of short fibrils is evident; these fibres are once again significantly shorter than those existing in the Fmoc-FRGDF hydrogel. Only discrete disordered aggregates were observed via AFM for Fmoc-PHSRN, suggesting that under these conditions, it did not form the desired nanofibrils (Figure 4g). We suggest that the proline residue perturbs the standard β-sheet conformation and subsequently stable fiber formation. This is in accordance with the observation of a blue shift shown in the CD spectra. The presence of these clusters is thought to contribute to the turbidity and inhomogeneity of the hydrogel. The hydrogel formed by the Fmoc-FRGDF/Fmoc-PHSRN peptide mixture showed a highly branched interpenetrating nanofibrous network comparable to that of the Fmoc-FRGDF gel alone.

**Figure 5:**
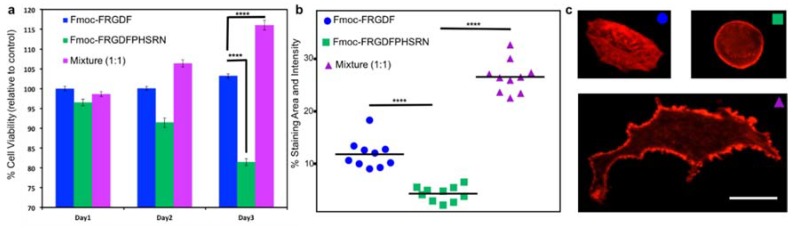
(**a**) Cell viability of HMFCs on peptide hydrogels. HMFCs were cultured on hydrogels containing peptides Fmoc-FRGDF, Fmoc-FRGDFPHSRN and peptide mixture (coassembled Fmoc-FRGDF/Fmoc-PHSRN) for three days. MTS assay was performed and the absorbance values were obtained at a wavelength of 490 nm. Absorbance value of cells grown on Fmoc-FRGDF was considered as control. A *p*-value of <0.05 was considered significant. (**b**) Analysis of spreading area: HMFCs were cultured on hydrogels containing peptides for 48 h. Cells were stained with actin and the images were obtained using fluorescent microscope (20× magnification). ImageJ analysis was performed and the values were plotted in a graph using GraphPad Prism, the *p*-value **** <0.05 was considered statistically significant. (**c**) Actin staining of HMFCs growing on hydrogels shows typical morphologies for each condition measured in (**b**) scale bar 10 µm.

Once the mechanical and chemical characteristics of all the peptide hydrogels are well known, 3D cell culture was used to determine the biological effects of the three systems. Fmoc-PHSRN was excluded as the peptide solution was unable to form a mechanically suitable hydrogel. All hydrogels were allowed to stabilize and form for 10 days. Human mammary fibroblast cells (HMFC) were grown via seeding on the surface of the three hydrogels (each containing either Fmoc-FRGDF (*R*), Fmoc-FRGDFPRHSRN (*RP*) or Fmoc-FRGDF/Fmoc-PHSRN (*R* + *P*)). HMFCs attachment and spreading on hydrogels containing *R*, *RP* and *R* + *P* were observed using actin staining at 48 h in order to allow the cells to establish and react to the various microenviroments. Cells cultured on the Fmoc-FRGDF hydrogels demonstrated viable cells, with a normal fibroblast structure. Conversely, the cells grown on Fmoc-FRGDFPHSRN were rounded, indicative of less spreading which can be attributed to lower attachment. Representative cells showed more significant spreading on the hydrogels containing a combination of Fmoc-FRGDF and Fmoc-PHSRN (Figure 5c). Further ImageJ analysis revealed significant increase in the area and staining intensity of the cells growing on this hydrogel in comparison to Fmoc-FRGDF and Fmoc-FRGDFPHSRN hydrogels (Figure 4). Research has shown that PHSRN and RGD are separated by 30–40 Å, this distance is important as PHSRN plays a synergistic role in cell adhesion and spreading [43], therefore the way in which PHSRN and RGD sequences interact within the system may affect cell adhesion and spreading in these materials.

Cell proliferation was determined using a colorimetric based MTS assay method. Cells grown on *R* gel tested on Day 1 were considered as the experimental control, as we have previously demonstrated its suitability as an attachment based cell culture scaffold [30]. The assay showed significant reduction in the viability of the cells grown on RP hydrogels, while a significant increase in cell viability was evident in *R* + *P* hydrogels after 3 days when compared to the control, indicating that the environment was beneficial to cell survival. Furthermore, the mixture of Fmoc-FRGDF/Fmoc-PHSRN showed a higher biocompatibility compared to that of Fmoc-FRGDFPHSRN (Figure 5). The low cell viability may due to the weak driver of assembly and insufficient attachment of HMFCs with the PHSRN and RGD domains localized on a single peptide.

## 4. Conclusions

Fmoc-FRGDF, as expected, self assembled to yield a nanofibrous network and hydrogelation. It’s synergistic epitope, Fmoc-PHSRN, was unable to assemble possibly due to the conformational constraint of the proline residue in the minimalistic sequence; PHSRN incorporation into a long peptide sequence, Fmoc-FRGDFPHSRN, gave rise to a weak gel, with nondesirable properties. Therefore, mixing minimalist SAPs improved the properties of gels in respect to both fibrillar nanostructure and material structural integrity. 3D cell cultures showed that while hydrogels containing one peptide epitope (RGD) can be used as an effective tool to mimic the 3D microenvironment of natural ECM, scaffolds formed via the coassembly of two different peptide signals (RGD and PHSRN) improved spreading, attachment and proliferation of cells. Overall, therefore, this work illustrates the importance of matching SAP properties to the application, and the design requirements of multicomponent self-assembled systems that can provide enhanced ECM mimicry for tissue engineering applications.
